# Supplementary material for: Complexome profiling of the Chlamydomonas psb28 mutant reveals TEF5 as an early PSII assembly factor
Source: Plant Cell. 2025 Mar 17;37(6):koaf055. doi: 10.1093/plcell/koaf055 (PMC12164590; doi:10.1093/plcell/koaf055)
Supplement: koaf055_Supplementary_Data [file koaf055_supplementary_data.zip › psb28_interactive.zip!/plots/intensity/qualityPlots/peptides/psbD_Cre-1.g2716961.t1.p1.html]

### Cre-1.g2716961.t1.p1

Trivial name: psbD  
SeqCoverage [median]: 33.20  
Mass: 39.45 kDa  
  
 

| Sequence | MissedCleavage | Mass [Da] | wtIntensity | muIntensity | Score |
| --- | --- | --- | --- | --- | --- |
| AAEDPEFETFYTK | 0 | 1546.68 | 2693872.11 | 571558.72 | 295.35 |
| AAEDPEFETFYTKNILLNEGIR | 1 | 2569.27 | 3035.01 | 0.00 | 63.69 |
| AFNPTQAEETYSMVTANR | 0 | 2028.92 | 490502.44 | 52307.67 | 252.05 |
| AWMAAQDQPHER | 0 | 1438.64 | 619908.49 | 76965.01 | 169.89 |
| AYDFVSQEIR | 0 | 1226.59 | 2108957.47 | 397874.35 | 281.09 |
| FWSQIFGVAFSNK | 0 | 1529.77 | 14317.57 | 0.00 | 209.19 |
| FWSQIFGVAFSNKR | 1 | 1685.87 | 5556.61 | 0.00 | 107.37 |
| LVFPEEVLPR | 0 | 1197.68 | 3668564.58 | 903294.55 | 229.33 |
| NILLNEGIR | 0 | 1040.60 | 2990247.63 | 608105.93 | 239.13 |
| QFEIAR | 0 | 762.40 | 911168.48 | 44864.04 | 187.93 |
| RTWFDDADDWLR | 1 | 1594.72 | 3850.73 | 0.00 | 97.55 |
| RTWFDDADDWLRQDR | 2 | 1993.90 | 923.34 | 0.00 | 30.54 |
| TIAIGTYQEK | 0 | 1122.59 | 421988.10 | 120056.54 | 223.08 |
| TIAIGTYQEKR | 1 | 1278.69 | 526702.74 | 207266.36 | 244.81 |
| TWFDDADDWLR | 0 | 1438.62 | 33812.66 | 0.00 | 159.34 |
| TWFDDADDWLRQDR | 1 | 1837.80 | 29872.40 | 0.00 | 100.19 |
|  |  |  | 14523280.4 | 2982293.2 |  |
